# Measurement Uncertainty Calculations for pH Value Obtained by an Ion-Selective Electrode

**DOI:** 10.3390/s18061915

**Published:** 2018-06-12

**Authors:** Józef Wiora, Alicja Wiora

**Affiliations:** Institute of Automatic Control, Silesian University of Technology, ul. Akademicka 16, 44-100 Gliwice, Poland; alicja.wiora@polsl.pl

**Keywords:** uncertainty evaluation, Monte Carlo simulation, potentiometry, pH measurement

## Abstract

An assessment of measurement uncertainty is a task, which has to be the final step of every chemical assay. Apart from a commonly applied typical assessment method, Monte Carlo (MC) simulations may be used. The simulations are frequently performed by a computer program, which has to be written, and therefore some programming skills are required. It is also possible to use a commonly known spreadsheet and perform such simulations without writing any code. Commercial programs dedicated for the purpose are also available. In order to show the advantages and disadvantages of the ways of uncertainty evaluation, i.e., the typical method, the MC method implemented in a program and in a spreadsheet, and commercial programs, a case of pH measurement after two-point calibration is considered in this article. The ways differ in the required mathematical transformations, degrees of software usage, the time spent for the uncertainty calculations, and cost of software. Since analysts may have different mathematical and coding skills and practice, it is impossible to point out the best way of uncertainty assessment—all of them are just as good and give comparable assessments.

## 1. Introduction

Exact knowledge of pH value is very important in many fields of engineering [1]. The value may be assayed using few methods, but the potentiometric method is still very popular. In this method, a glass ion-selective electrode (ISE), which converts the pH value in to an electrical signal, is used and the signal can be easily transformed by any modern control system.

Each result of the measurement should consist of both the quantity value and an indication of the quality of the measurement performed. The indication is necessary during the comparison of measurements among themselves or with references. Nowadays, the measurement uncertainty should be considered as the indication. It was formerly the measurement error, but since the exact values of errors are never known, it is not considered as the best representative of measurement quality [2].

Evaluation of uncertainties should be done according to the guide to the expression of uncertainty in measurement (GUM) [2] or the Quantifying Uncertainty in Analytical Measurement (QUAM) Guide [3], which chemists frequently reach for. The most important information is gathered in the following section of the GUM: ‘The principles of uncertainty calculations’. Unfortunately, the process can be time-consuming in some cases. Sometimes, very long mathematical expressions occur and the determination of uncertainties in a typical way is hindered. However, a computational method of uncertainty evaluation, called the Monte Carlo (MC) method, which facilitates the process exists. A computer program is usually written to apply the MC method, but not everyone is able to write a software. A technique of evaluating the uncertainties without programming and using one of the popular spreadsheets also exists. In the article, the approaches to uncertainty calculations are summarized, applied to the pH case, compared, and their strengths and weaknesses are highlighted. The goal of the work is to show ways of uncertainty calculations rather than an enumeration of all uncertainty sources during pH measurements.

## 2. Potentiometric Principles

To perform a potentiometric measurement, an ion-selective electrode and a reference electrode are immersed in an investigated solution. In the case of pH, both electrodes are often enclosed in one housing that makes the so-called pH combination electrode. The potential difference (voltage) *E* between the electrodes is measured using a high-impedance voltmeter. The simplest mathematical model of the measurement is the Nernst equation [4]:

(1) $$E=E^{\circ }+S\log\left(a_{B}\right),$$

where $E^{\circ }$ is the standard potential, *S* is the Nernstian slope, and $a_{B}$ is the activity of primary ions B. This model, often used in practice, does not take into account an effect of the so-called liquid junction potential at the interface between the reference electrode and the solution. In this work, in order to simplify considerations, the effect is omitted.

In the case of pH measurement, where the pH value is defined as

(2) $$\mathrm{pH}=-\log\left(a_{H}\right),$$

the relationship between the voltage and pH is as follows:

(3) $$E=E^{\circ }-S\cdot \mathrm{pH}.$$

Despite the fact that the parameters $E^{\circ }$ and *S* have their theoretical values [4,5], in practice, they drift as a result of continuous processes which take place inside the electrode, such as hydration of glass. Therefore, each ISE requires frequent calibration. To do it, in the simplest case, two buffer solutions of known pH are used. Potentials of the ISE, $E_{1}$ and $E_{2}$, are read sequentially after immersing the electrode in the first and in the second buffer whose pH values are ${\mathrm{pH}}_{1}$ and ${\mathrm{pH}}_{2}$, respectively. The electrode should be washed or rinsed in distilled water before immersions. After the experiment, the parameters may be calculated. Further potential measurements, with the electrode immersed in an unknown solution X, allow using Equation (3) to determine the unknown pH value as [6,7]

(4) $${\mathrm{pH}}_{X}={\mathrm{pH}}_{1}-\left(E_{X}-E_{1}\right)/\hat{S},$$

where

(5) $$\hat{S}=\frac{E_{1}-E_{2}}{{\mathrm{pH}}_{2}-{\mathrm{pH}}_{1}}.$$

Other calibration methods, such as multi-point calibration, can also be applied. Then, the parameters of the model may be calculated using least squares or least median of squares methods [7,8,9]. Contemporary instruments dedicated for pH measurement, called pH-meters or ion-meters, provide very-high input impedance measurement of electrode potentials and calibration procedures. The user has to assess the measurement uncertainty, however, by herself.

## 3. The Principles of Uncertainty Calculations

To determine uncertainties of measurements, it is necessary to specify the terminology associated with the problem. The most important information is included in an international standard—the GUM [2] and in the International Vocabulary of Metrology (VIM) [10]. The standards are based on the uncertainty approach. Nevertheless, the classical terminology based on the classical approach, such as a systematic and random error, is still used. Therefore, the correct distinction between the Type A and Type B evaluations of measurement uncertainty and the way in which the result of measurement should be reported are very important.

Term error of measurement used in the classical approach is contemporarily interpreted as a difference between an individual measured value $x_{i}$ and the reference (or true) value of the measurand and is usually denoted by the symbol $\Delta x_{i}$. The error is not an object of interest of the uncertainty calculation since a priori calculation is not possible—it is an idealized concept and the error cannot be known exactly. It is only a single representation of a random variable. The value of a known error can be applied as a correction to the measured value [3].

The uncertainty of measurement is a “non-negative parameter characterizing the dispersion of the quantity values being attributed to a measurand, based on the information used” [10]. All input estimates (e.g., input quantities of the measurement model, and its parameters) are sources of uncertainties that contribute to the uncertainty of the measured value. Therefore, the uncertainties of the sources should be evaluated according to either the Type A or the Type B method.

The Type A evaluation of standard uncertainty is the method using the statistical analysis of a series of observations and is performed after experiments. The uncertainty is estimated as the experimental standard deviation of the mean that follows from an averaging procedure or an appropriate regression analysis. This “can be applied when several independent observations have been made for one of the input quantities under the same conditions of measurement”. In such a situation, a scatter (or spread) in the obtained values is observable [11]. If the scatter of the experimentally determined values is small, then the precision is high. A quantitative measure of the scatter is the standard deviation of a set of measurements. Therefore, if the precision is high, then the standard deviation is small. It is important to recognize that the statistical measure of the quantity of an assay cannot be obtained unless a series of tests is carried out [12].

According to the GUM, different probability distribution in the uncertainty evaluation can be applied. In analytical chemistry, the Laplace–Gauss (also known as Gaussian or normal) distribution is usually adequate [13]. It is characterized by the following parameters: σ—the standard deviation or $\sigma^{2}$—the variance and μ–a mean that would result from an infinite number of measurements. The parameters can be calculated from:

(6) $$\mu =\lim_{N\to \infty }\frac{1}{N}\sum_{i=1}^{N}x_{i},\;\sigma^{2}=\lim_{N\to \infty }\frac{1}{N}\sum_{i=1}^{N}{\left(x_{i}-\mu \right)}^{2},$$

where *N* is the total number of elements in the population, which should be infinite. The Gaussian distribution describes the common and general mathematical model of the distribution of analysis results. However, it can only be applied in the range of μ±3σ [14].

It is, however, impossible to make an infinite number of assays. Thus, it is also impossible to know the values of μ and σ. In such a situation, only estimates of the parameters can be calculated, preceded by a series of experiments. The chosen measured samples have to be the representative of the population [13].

A very effective assessment of the parameter μ (i.e., characterized by a minimal scatter of estimates around the parameter) is the arithmetic mean $\bar{x}$:

(7) $$\bar{x}=\frac{1}{N}\sum_{i=1}^{N}x_{i},$$

often simply called the mean or the average [12]. However, the mean is sensitive to any asymmetry of a distribution or to gross errors in the case of a limited number of measurements. Then, the median is a better estimate [14,15].

There exist some estimates of σ. Frequently, it is assessed according to [11,14,16]:

(8) $$s\left(x\right)=\sqrt{\frac{1}{N-1}\sum_{i=1}^{N}{\left(x_{i}-\bar{x}\right)}^{2}},$$

where N>1, s(x) is the experimental standard deviation and $s^{2}\left(x\right)$ is the experimental variance of values $x_{i}$.

When a finite number of elements is available, the mean $\bar{x}$ is only an estimate of μ. If the individual values of $x_{i}$ have a Gaussian distribution that are independent and their variances are equal to each other, then the variance of the mean is *n*-times smaller. This implies that the experimental standard deviation of the mean $s(\bar{x})$ in a series of *N* determinations is related to the experimental standard deviation for a single value in the series s(x) by [11,14]:

(9) $$s\left(\bar{x}\right)=\frac{s(x)}{\sqrt{N}}.$$

This approach is not always sufficient during the calculation of a measurement result. In the general case, the probability distribution of a single measurement and of the mean is not the same. The values of a single measurement may be described by different distributions. According to the Central Limit Theorem and the Law of Large Numbers, the shape of the distribution of a mean depends on the number of measurements in the series, At the limit, for N→∞, the distribution of the mean approaches the normal distribution [17].

The Type A evaluated standard uncertainty $u_{A}$ associated with the estimate $\bar{x}$ is the experimental standard deviation of the mean [11]:

(10) $$u_{A}\left(x\right)=s\left(\bar{x}\right).$$

In the analytical practice, too many parallel assays are usually not performed as they are too tedious. Therefore, the long-lasting analytical procedure does not allow analysts to perform more than 2–3 assays of the same probe [14].

The Type B evaluation of standard uncertainty is a method performed using means that are different from the statistical analysis of a series of observations, which is based on some other scientific knowledge, and may be assessed a priori [11]. It utilizes the available information on the possible variability of a quantity. The information may come from previous measurements, calibrations, general knowledge, manufacturer’s specifications, or handbooks and the uncertainty component can be calculated before experiments [11,18].

The next very important aspect is the propagation of uncertainties. In the classical approach, a measurand can be described by a single true value, but instruments and measurements do not provide this value due to the additive errors: systematic and random. These errors have to be treated differently in an error propagation and it is assumed that they may always be distinguished. In the uncertainty approach, there is only one uncertainty of measurement, ensuing from various components. It characterizes the extent to which the unknown value of the measurand is known after the measurement, taking into account the given information from the measurand [10].

If a mathematical model of a measurement, $y=f(x_{1},x_{2},\ldots ,x_{M})$, can be expressed as a sum or as a product, the propagation of uncertainty is relatively simple to calculate. Such a didactic example is presented by Olson and Sattar [19]. If the model is more complicated, advanced calculations should be applied.

For uncorrelated input quantities and when the function *f* is not strongly nonlinear, the square of the combined standard uncertainty, $u_{c}$, associated with the output estimate *y* is given by [2,11]:

(11) $$u_{c}^{2}\left(y\right)=\sum_{j=1}^{M}u_{j}^{2}\left(y\right).$$

The quantity $u_{j}\left(y\right)$ is the contribution of the standard uncertainty associated with the output estimate *y*, resulting from the standard uncertainty associated with the input estimate $x_{j}$ calculated from

(12) $$u_{j}\left(y\right)=c_{j}u\left(x_{j}\right),$$

where $c_{j}$ is the sensitivity coefficient associated with the input estimate $x_{j}$:

(13) $$c_{j}=\frac{\partial f}{\partial x_{j}}.$$

Some simplification in evaluation of combined uncertainty is proposed in the work of Kušnerová et al. [20].

For the correlated input quantities, calculation of the combined standard uncertainty is much more complicated. It is necessary to take into account the correlation coefficients between all the correlated input quantities. An excellent tutorial is included in Appendix D of EA-4/02 [11]. Problems with correlations in potentiometric measurements are considered in other works [7,21].

The expanded uncertainty is an additional measure of uncertainty, denoted by *U*, obtained from multiplying the combined standard uncertainty by a coverage factor *k*. It allows one to express the result of the measurement as y±U. The *y* is the mean that gives the best estimates of the value attributed to the measurand. Most of the values that could reasonably be attributed to the measurand are included in the interval defined by (y−U) to (y+U) [2]. It has been widely accepted that the value of the coverage factor *k* should correspond to the coverage probability (also known as the confidence level) of approximately 95 %. Then, *k* is approximately equal to 2 [11].

In general, uncertainty of the output quantity can be read from a probability density function (PDF) of the quantity. In Supplement 1 of GUM, the following implementations of the propagation are quoted [22]:

1. The analytical method. It requires very good skills in mathematical transformations of PDF and is therefore very time-consuming.
2. The first-order Taylor approximation method. It is based on replacing the model by a first-order Taylor series. It is the typical method for uncertainty evaluating and is very often applied.
3. The *n*th order Taylor approximation method. It is based on replacing the model by a higher-order Taylor series. The method is more exact, especially for nonlinear models, but requires more advanced mathematics. The possibility of making mistakes during the mathematical transformations is high.
4. The numerical method. It implements the propagation of PDF, is very effective, but requires better programming skills.

This paper refers only to Methods #2 and #4.

## 4. Materials and Methods

A hypothetical pH measurement of tap water was considered in this paper. A typical combined pH electrode was calibrated with ${\mathrm{pH}}_{1}=4$ and ${\mathrm{pH}}_{2}=9$ buffers. After calibration, the electrode was immersed in a sample X. Each potential reading was conducted five times. The indications are gathered in Table 1.

The dispersion seen may result from, e.g., disturbances influencing the electrical circuit. Additionally, the tolerances of pH buffers were known as ±0.05 pH, as from the Avantor Performance Materials Poland S.A. (Gliwice, Poland). The tolerances of the pH-meter displaying voltage was ±0.3 mV, comparable with the ORION Model 930 Ionalyzer (Orion Research, Cambridge, MA, USA). The tolerance of pH delimits the interval in which the real value of pH is and the tolerance of potential provides information about how the indication would differ from the one obtained by a hypothetical perfect meter.

## 5. Results and Discussion

The measurement result is elaborated in this section. In order to do it, the pH value is calculating first, based on calibration data, and its measurement uncertainty is evaluated afterwards using different ways.

### 5.1. Calculation of Unknown pH Value

The electrode slope is obtained from Equation (5), in which mean values of potentials seen in Table 1 are used. The value

$$\hat{S}=\frac{182.4\;\;\mathrm{mV}-(-103.8\;\;\mathrm{mV})}{9-4}=57.24\;\;\mathrm{mV}.$$

It is also possible to obtain the standard potential by the appropriate transformation of Equation (3) to

$${\hat{E}}^{\circ }=E_{1}+\hat{S}\cdot {\mathrm{pH}}_{1}=182.4\;\;\mathrm{mV}+57.24\;\;\mathrm{mV}\cdot 4=411.36\;\;\mathrm{mV}.$$

The unknown pH value is calculated using the following Equation (4) recommended by the International Union of Pure and Applied Chemistry:

(14) $${\mathrm{pH}}_{X}=4-\frac{9.3\;\;\mathrm{mV}-182.4\;\;\mathrm{mV}}{57.24\;\;\mathrm{mV}}=7.024109.$$

### 5.2. Uncertainty Evaluation

The process of the uncertainty determination is not very simple in general. The GUM recommends some approaches including the typical Taylor approximation and Monte Carlo (MC) method. The MC requires a computer program to simulate probability distributions; therefore, some program has to be written. It is also possible to perform the simulation using a popular spreadsheet. Commercial software is also available that provides a very comfortable way of uncertainty determination. The uncertainty evaluation using the Taylor approximations, computer programs, spreadsheets and commercial software are characterised below.

#### 5.2.1. Method with the Taylor Approximation

This is the most popular method and other popular documents, such as QUAM Guide [3], EA-4/02 document [11], and A2LA Guide [23], refer to it. For this reason, the method is named typically in this paper. The process of uncertainty calculation is complicated; therefore, it is good to split it into some stages that are enumerated below.

Stage 1. Determination of sources

In the considered example, there are five input quantities: $E_{1},E_{2},E_{X},{\mathrm{pH}}_{1}$, and ${\mathrm{pH}}_{2}$. The quantities are uncorrelated among themselves. It is very convenient to collect their standard uncertainties.

The considered experiment contains five indications of potentials to each solution. It allows for the calculation of Type A evaluated uncertainties. Applying Equations (8)–(10) with *n* = 5, the following is obtained:

(15) $$u_{A}\left(E_{1}\right)=\frac{1}{\sqrt{5}}\sqrt{\frac{1}{4}\sum_{i}{\left[\Delta E_{1}\left(i\right)\right]}^{2}}$$

and using the data from Table 1, the uncertainty is

(16) $$u_{A}\left(E_{1}\right)=\sqrt{\frac{1}{5}\cdot \frac{1}{4}\cdot 26\cdot {\left(10^{-1}\;\;\mathrm{mV}\right)}^{2}},$$

(17) $$u_{A}\left(E_{1}\right)=1.14\cdot 10^{-1}\;\;\mathrm{mV}=0.114\;\;\mathrm{mV}.$$

In this place, it is worth noting that the uncertainty may be written with a higher precision, but, from a practical point of view, such intermediate uncertainties are better to be written with three or four significant figures. Higher precision does usually not transform to better uncertainty assessment because the combined uncertainty has to be rounded off.

In the same way, Type A evaluated uncertainties of $E_{1}$ and $E_{X}$ are obtained:

(18) $$u_{A}\left(E_{2}\right)=\sqrt{\frac{1}{5}\cdot \frac{1}{4}\cdot 10\cdot {\left(10^{-1}\;\;\mathrm{mV}\right)}^{2}}=0.0707\;\;\mathrm{mV},$$

(19) $$u_{A}\left(E_{X}\right)=\sqrt{\frac{1}{5}\cdot \frac{1}{4}\cdot 20\cdot {\left(10^{-1}\;\;\mathrm{mV}\right)}^{2}}=0.100\;\;\mathrm{mV}.$$

Potentials are measured with a tolerance of ±0.3 mV. No additional information is given, thus the distribution is unknown. For such a case, GUM recommends calculating the Type B component as the tolerance divided by $\sqrt{3}$. It gives

(20) $$u_{B}\left(E\right)=0.3\;\;\mathrm{mV}/\sqrt{3}=0.173\;\;\mathrm{mV}.$$

Now, two uncertainty sources are given: (1) arising from the repetitive measurements and (2) from the nonideality of the meter used. No complicated mathematical model exists–both sources influence the result in the same way, so the two contributions should be joined together geometrically. The obtained combined uncertainty of $E_{1}$ is calculated from

(21) $$u_{c}\left(E_{1}\right)=\sqrt{uA^{2}\left(E_{1}\right)+u_{B}^{2}\left(E\right)}$$

as

(22) $$u_{c}\left(E_{1}\right)=\sqrt{{\left(0.114\;\;\mathrm{mV}\right)}^{2}+{\left(0.173\;\;\mathrm{mV}\right)}^{2}}=0.207\;\;\mathrm{mV}.$$

In the same way,

(23) $$u_{c}\left(E_{2}\right)=\sqrt{{\left(0.0707\;\;\mathrm{mV}\right)}^{2}+{\left(0.173\;\;\mathrm{mV}\right)}^{2}}=0.187\;\;\mathrm{mV}$$

and

(24) $$u_{c}\left(E_{X}\right)=\sqrt{{\left(0.100\;\;\mathrm{mV}\right)}^{2}+{\left(0.173\;\;\mathrm{mV}\right)}^{2}}=0.200\;\;\mathrm{mV}$$

are calculated.

Regarding the pH buffers, no validation of their values were performed and no statistical analysis is done, so only the Type B evaluation may be conducted. Applying tolerances, the uncertainties for the two buffers are the same and are calculated as

(25) $$u_{B}\left(\mathrm{pH}\right)=0.05/\sqrt{3}=0.0289.$$

Stage 2. Determination of measurement model

Here, the model of measurement is described by Equations (4) and (5). Unfortunately, evaluation of uncertainty using the equations is complicated due to existing correlations—both equations consist $E_{1}$. An approach with the evaluation of uncertainties in two steps, first of parameters $\hat{S}$ and ${\hat{E}}^{\circ }$ and further of the seek ${\mathrm{pH}}_{X}$, is also complicated as it is proven elsewhere [7]. For this reason, the most convenient model for uncertainty evaluations looks like:

(26) $${\mathrm{pH}}_{X}={\mathrm{pH}}_{1}-\frac{E_{X}-E_{1}}{E_{1}-E_{2}}\left({\mathrm{pH}}_{2}-{\mathrm{pH}}_{1}\right).$$

Stage 3. Derivation of sensitivity coefficients formulae

It is necessary to derive the sensitivity coefficients according to Equation (13), symbolically, by making the derivation of Equation (26) with respect to all sources. The task does not have to be simple, in general. It is possible to apply specialized software, like Mathcad (MathSoft, Cambridge, MA, USA) or MATLAB (Mathworks, Sherborn, MA, USA). Numerical calculations are also allowed [11], but the values of the coefficients change with the change of sources and it is necessary to recalculate them for each example.

Here, the sensitivity coefficient formula for the potential indications of the pH-meter when the electrode is immersed in the first buffer is:

(27) $$c_{E_{1}}=\frac{\partial {\mathrm{pH}}_{X}}{\partial E_{1}}=\frac{\left(E_{2}-E_{X}\right)\left({\mathrm{pH}}_{1}-{\mathrm{pH}}_{2}\right)}{{\left(E_{1}-E_{2}\right)}^{2}}$$

and in the second buffer:

(28) $$c_{E_{2}}=\frac{\partial {\mathrm{pH}}_{X}}{\partial E_{2}}=\frac{\left(E_{X}-E_{1}\right)\left({\mathrm{pH}}_{1}-{\mathrm{pH}}_{2}\right)}{{\left(E_{1}-E_{2}\right)}^{2}}.$$

The sensitivity coefficient for indications obtained during the measurement of the sample is

(29) $$c_{E_{X}}=\frac{\partial {\mathrm{pH}}_{X}}{\partial E_{X}}=\frac{{\mathrm{pH}}_{1}-{\mathrm{pH}}_{2}}{E_{1}-E_{2}}.$$

The coefficients for pH values of buffer solutions are

(30) $$c_{{\mathrm{pH}}_{1}}=\frac{\partial {\mathrm{pH}}_{X}}{\partial {\mathrm{pH}}_{1}}=\frac{E_{X}-E_{2}}{E_{1}-E_{2}}$$

and

(31) $$c_{{\mathrm{pH}}_{2}}=\frac{\partial {\mathrm{pH}}_{X}}{\partial {\mathrm{pH}}_{2}}=\frac{E_{1}-E_{X}}{E_{1}-E_{2}}.$$

Stage 4. Building the budget

Now, it is time to begin building the uncertainty budget. Because the input quantities are not correlated, the combined standard uncertainty can be calculated according to Equations (11) and (12). Using one of the popular spreadsheets is very efficient. It is recommended to organize the budget as in Table 2 [11].

The first rows contain headers. The rows ‘Sources’ contain the main calculations. In the first two columns, the quantity symbols and their estimates are presented. Column (3) of Table 2 contains the standard uncertainties, which are determined in Stage 1. The next column, (4), contains the values of the sensitivity coefficients obtained in Stage 3. Using a spreadsheet, cells of the column should contain appropriately rewritten formulae, Equations (27)–(31), according to the spreadsheet standards. The formulae are much more readable when so-called named cells are applied. Column (5), uncertainty contribution, is derived from multiplications of Column (3) by Column (4). Column (6) is the square of Column (5). Finally, in the ‘Result’ row, all the squares are summed. The combined standard uncertainty of the measured pH is the root of the previously calculated sum.

Stage 5. Reporting the result

The calculated standard uncertainty of the pH is 0.02131. The expanded uncertainty is 0.04262 and follows from the multiplication of the value by a coverage factor, which is two. Because the reported uncertainty should have no more than two significant figures, the obtained value should be rounded [11]. Finally, the estimate of measurement value should have the same number of digits after the decimal point as the uncertainty. Therefore, the obtained result of measurement is

(32) pH=7.024±0.043.

The grading of the quality of the pH measurement depends on the application and is dependent on its measurement span. Whereas expanded uncertainty of ±0.043 pH units is very good for sewage where the typical pH ranges from 6 to 9, it is poor for arterial blood with the typical range of 7.35 up to 7.45.

The typical method for evaluating the uncertainty with a budget written in tabular form is very transparent. Indicating the source, which gives the greatest contribution is very simple, here it is ${\mathrm{pH}}_{2}$. This makes it possible to improve the measurement by decreasing the uncertainty in the future, in this case, by purchasing a better standard buffer with a lower tolerance. No expensive tools are required, since spreadsheets are available as freeware and most computer users are familiar with them. However, the method requires the derivations to be solved. It is possible to make some mistakes during the process, therefore it should be done carefully. Creating the spreadsheet formulae for sensitivity coefficients may be troublesome, particularly if the equations are long and complicated.

#### 5.2.2. Numerical Method Implemented in a Program

This method consists of the Monte Carlo simulations, in which the propagation of distribution is performed by random sampling from the probability distribution. It is an approach that has become more and more popular with the development of computers. An application of the method to error propagation was described in a bygone article by Douglas [24]. The author performed simulations in BASIC and used only a set of 44 data as a compromise between the computer time required and the precision. Now, a set of thousands of data can be calculated in an imperceptible time as it is proven in new publications [25,26,27,28]. The method is especially useful if the linearization of the model provides an inadequate representation, and may sometimes be easier to apply due to difficulties in calculating the sensitivity coefficients [22].

**Figure 1:**
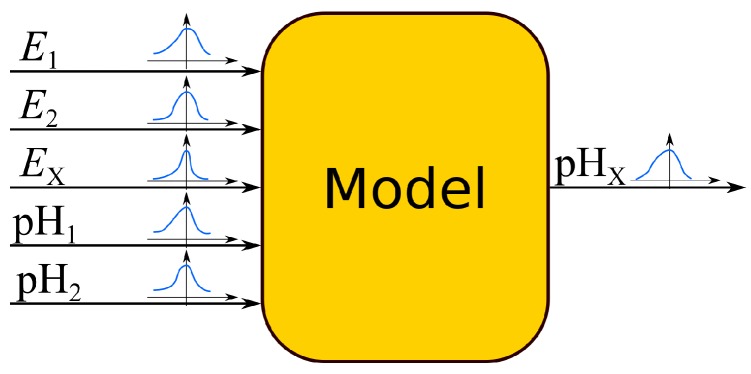
Concept of the Monte Carlo method.

The idea of the MC method is very simple. Input quantities are described by mean values and PDFs. By applying simulations, it is possible to generate a set of data where random numbers are added to the means. The random numbers have assumed distributions with standard deviations equal to the standard uncertainties. Next, a set of output values is calculated using the measurement model. At the end, the output distribution is analyzed and its standard deviation is calculated, which is equal to the standard uncertainty of the output quality. Figure 1 illustrates the example.

Uncertainty evaluation consists of stages similar to these conducted by the typical method, except that Stages 3 and 4 are replaced by the mentioned simulations. To assess the combined uncertainty of the output quantity, it is necessary to apply some computer program. Such program may look like that written in C++ and presented in Appendix A. After initialization and model definition, a loop is performed, in which random variables are generated and added to the parameters of the model. Then, the pH value is calculated, compared with the true value and the error is squared and cumulated. When the loop is finished, the uncertainty is calculated as the square root of cumulated squared errors divided by the number of loops performed. Such approach engages a small amount of memory and works quite fast.

Such simulations may also be performed writing code with very compact matrix notations. The code presented in Appendix B is written in the MathWorks^®^ MATLAB language, which is very often used by scientists in developing the MC method [25]. The script works well also in the freeware implementation—GNU Octave. It consists of initialization of variables with measurement values and uncertainties, the definition of the model function, the definition of vectors with disturbed quantities, calculation of the vector with disturbed measured pH, and finally the calculation and presentation of standard deviation. A similar code presented in Appendix C is written in the R language. The language is very popular among statisticians and is distributed as freeware. The script is very similar that written in Matlab. It is also possible to write code using matrix notation in a general-purpose programming language, such as Python. The example is included in Appendix D. Application of the Numpy library makes the code very similar to the Matlab and R ones.

**Figure 2:**
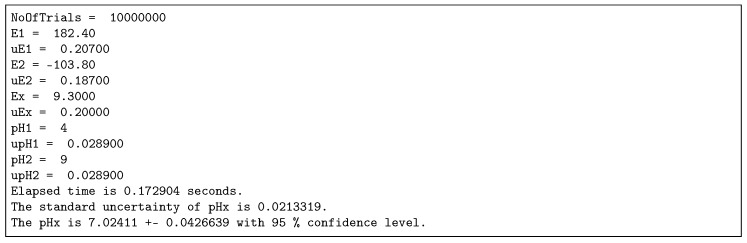
Outputs of the Monte Carlo simulations obtained by the script run in GNU Octave.

The outputs of the programs may look like that presented in Figure 2.

The simulation times performed using the enumerated languages differs a bit—cf. Table 3.

The differences are caused rather by the complexity of the algorithm of the random number generator applied in the scripts than the optimisation of the code.

The MC simulations are performed very fast. Uncertainties obtained by simulations are different from simulation to simulation. This is an inherent feature of the method. However, if the number of trials is in the order of $10^{6}$, the results differ among themselves by about 0.1%. If the number of trials is higher, the uncertainty is predicted better, but the program takes longer to calculate. The probable error varies inversely with the square root of the number of trials [29].

The use of the MC method makes it possible to analyze the shape and asymmetry of the output PDF resulting from the propagation through a function, which can be nonlinear. The typical propagation method uses linearization, and therefore the effect is invisible [25].

During the evaluation of measurement uncertainty by applying the MC simulations implemented in a program, it is not required to solve derivatives. Instead, programming skills are needed. The obtained uncertainty is not exact, but it is very close. In some complicated and nonlinear cases, the MC result can be considered as more reliable than the result obtained from the typical propagation method [25]. A computer program that executes calculations, like GNU Octave, has to be installed or its online implementation may be applied.

#### 5.2.3. Numerical Method Implemented in a Spreadsheet

According to observations of the authors, the ability to work with spreadsheets is a much higher skill than the programming skills. For this reason, an implementation of the numerical method to a spreadsheet omitting the programming is worth attending.

In the literature, the applications of spreadsheets for the MC calculations are known. Excel (Microsoft, Redmond, WA, USA) has been used, among others, for predicting the reliability parameters by Gedam and Beaudet [30], and for the evaluation of uncertainties in grating the pitch measurement by Decker et al. [31]. However, they have applied macros that require some programming skills. In another work, the MC simulations have been performed using Excel to estimate uncertainties in the ecosystem budget calculations, but the results of each trial have been manually copied into a separate spreadsheet [32]. There also exist some commercial spreadsheet-based applications, like Crystal Ball (Oracle, Redwood Shores, CA, USA), which support MC simulations.

It is possible to evaluate the uncertainties using one of the popular spreadsheets in a very convenient way. It uses the MC method, but no program is written. Therefore, it does not require any programming skills. Such approach is known from a conference presentation [33] and from other papers [34]. To obtain the uncertainty, one should perform the Stages 1 and 2 in the same way as in the typical method. Thereafter, the MC simulations should be implemented in the way described below in three steps. Finally, the measurement results should be reported as in Stage 5.

Step 1. Entering the input data

First of all, the input data should be entered into the spreadsheet.

**Figure 3:**
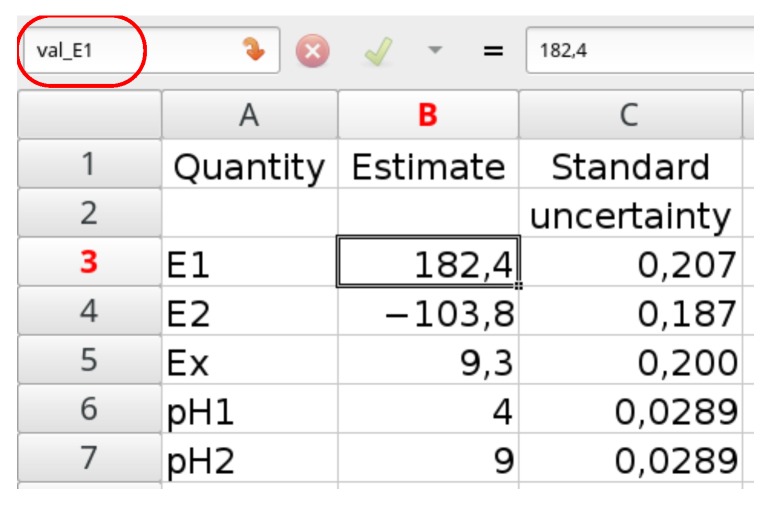
A table in a spreadsheet containing the input data and their uncertainties.

It is very convenient to organize it as in Figure 3: the first column consists of quantity symbols, the second consists of estimates, and the third is of their standard uncertainties. Additionally, using named cells, the formulae are more human readable. Here, the $B$3 cell containing the value of $E_{1}$ is named as val_E1 and the $C$3 cell containing its uncertainty is named as un_E1. The remaining cells are named in the same way.

Step 2. Generation of uncertain input data

To generate random variables with standard normal distribution, the =NORM.S.INV(RAND()) formula should be used in the spreadsheet. The GUM allows for treating all uncertainty contribution, as if the distributions were Gaussian [35]. Creating a spreadsheet causes an inconvenience occurs—a change of commands with time between spreadsheets. Older Excel versions, Gnumeric and Google Sheets use commands without dots, such as NORMSINV(), which are treated as deprecated in newer versions of Excel. For the above reason, the user has to decide whether the code should be universal or up-to-date.

Additionally, most of the available spreadsheets are nationalized. This means that the names of the functions are translated to the default language used in the operating system. Therefore, the same function in Polish looks like =ROZKAD.NORMALNY.S.ODW(LOS()) by deprecated syntax or =ROZKŁ.NORMALNY.S.ODWR(LOS()) using modern syntax. It is possible to find the function name translations on the Internet [36].

**Figure 4:**
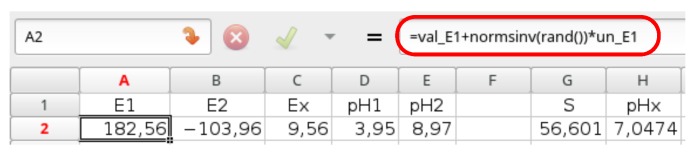
A table in Gnumeric containing the uncertain input data.

In the presented example, a table with five columns with five corresponding input quantities and a few thousand rows of random numbers should be generated, and it is recommended in a separate sheet. The number of rows corresponds to the number of trials. To do that, it is necessary to write the appropriate formulae in the first row describing the uncertain input quantities, e.g., the A2 cell may contain formula corresponding to $E_{1}+\xi \cdot u\left(E_{1}\right)$, where ξ is a normalized Gaussian random variable in the form with the theretofore defined named cells: =val_E1+NORM.S.INV(RAND())*un_E1—see Figure 4.

Now, it is recommended to name columns, e.g., the columns A:A as _E1 (referencing to named columns does not work well in LibreOffice). It allows for writing the formulae describing Equations (4) and (5) in the human-readable form as: =(_E1-_E2)/(_pH2-_pH1) for the slope and =_pH1-(_Ex-_E1)/_S for the measured pH, respectively. After preparation of the first row containing appropriate formulae, here, the row 2:2 should be copied many times, ideally thousands of times. It can be done by the drag-down technique, which may be ineffective here. It is better is to select the first data row and, while holding down the SHIFT key, press PAGE DOWN key many times. It is also possible to apply the CTRL+G keyboard shortcut, but it works in a different way, depending on the spreadsheet used. After selecting the appropriate region, the CTRL+D shortcut duplicates the formulae.

Step 3. Uncertainty calculation

**Figure 5:**
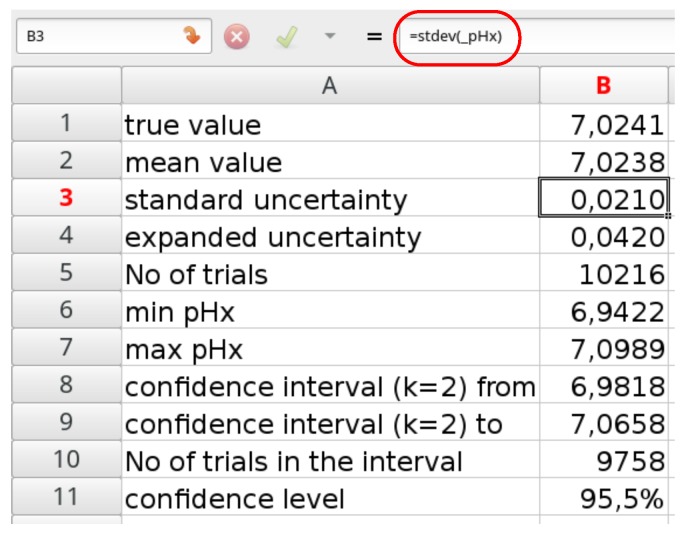
Output data calculated in Gnumeric.

The last step consists of calculations of the statistical parameters. The most important is the standard uncertainty. The best way is to use a separate sheet and calculate it there using =STDEV.S(_pHx) formula. Other parameters may also be calculated, as it is illustrated in Figure 5.

The standard uncertainty calculated using MC simulation is close to that obtained using the typical method shown in Table 2.

The presented way of implementing the MC method works both on Microsoft Excel and on freeware spreadsheets such as Gnumeric, Google Sheets and partially on LibreOffice Calc. Knowledge of spreadsheets is probably much greater than the knowledge of computer programming. No compilation is required, as the result appears immediately after the data has been entered. The time required for the preparation of the uncertainty calculation tool is the shortest for most users. If there are too many trials, the results can appear after some time, depending on the computer capability.

#### 5.2.4. Uncertainty Evaluation Using Dedicated Software

There are a lot of dedicated software for evaluation of measurement uncertainties. Such list is available on Wikipedia [37].

**Figure 6:**
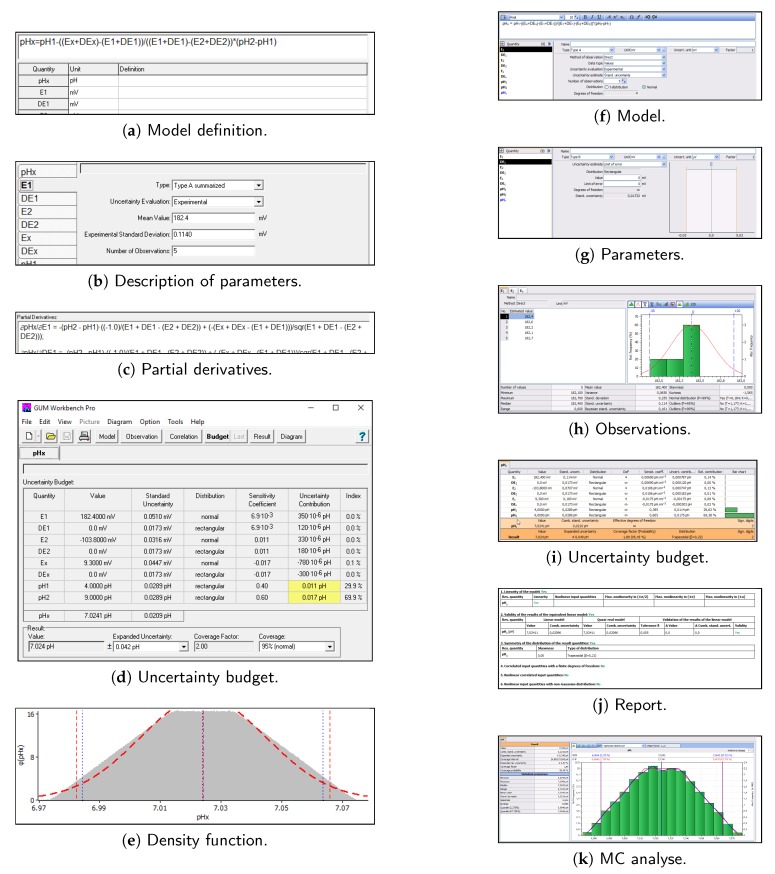
Screens of Metrodata GUM Workbench Professional Version 2.4 (**left**) and Qualisyst GUM Enterprise 4.10 (**right**).

GUM Workbench (Professional Version 2.4, Metrodata, Braunschweig, Germany) is a commercial software program [38]—the single user licence is 3213 EUR. A demo version that does not allow for data saving is available for free. The application has a friendly and intuitive user interface (UI). It allows for introducing a measurement model (Figure 6a), and their parameters with values, units, uncertainties and distributions (Figure 6b). The sensitivity coefficients are calculated symbolically (Figure 6c). The budget is built and dominant sources are pointed out (Figure 6d). The measurement result is reported according to the standard. Additionally, Monte Carlo simulations may be performed to show the resulting PDF shape (Figure 6e).

Similar functions offer GUM Enterprise (version 4.10, Qualisyst, Gabrovo, Bulgaria) [39]. Single-user licence is 1250 EUR. Simplified, non-commercial, academic, trial and demo versions are also available. We cannot find formulae of partial derivatives; instead, there are a lot of statistical parameters. Screens taken from the software are presented in Figure 6f–k.

Commercial programs guarantee high reliability of correctness of the obtained calculation results. They are easy to use and provide a lot of additional statistical parameters. Their main disadvantage is the price, which can be a problem for some users.

## 6. Conclusions

The uncertainty evaluation process is time-consuming. The typical method recommended by GUM requires finding the derivatives of the measurement model, which may have a complicated form. The Monte Carlo method applies simulations and avoids the disadvantage—the propagation of probability distribution is analyzed numerically. The simulations are usually realized in a computer program, which requires some programming skills. It is also possible to perform the simulations in a commonly applied spreadsheet. Thus, no program is required, but some formulae describing the measurement model and simulations have to be entered. In this way, the task of uncertainty calculations is done very fast. The MC simulations can facilitate uncertainty evaluation, especially when the model describing the phenomenon has a complicated form. Commercial applications dedicated for uncertainty calculations are also available but are not cheap. The obtained assessments of the uncertainty are very similar to each other. All of the approaches have their pros and cons, and therefore should be chosen depending on the user skills and practice.

## Figures and Tables

**Table 1:** Potential indications of pH-meter during calibration and measurement.

No	$E_{1}$	$\Delta E_{1}$	${\left(\Delta E_{1}\right)}^{2}$	$E_{2}$	$\Delta E_{2}$	${\left(\Delta E_{2}\right)}^{2}$	$E_{X}$	$\Delta E_{X}$	${\left(\Delta E_{X}\right)}^{2}$
*i*	mV	$10^{-1}$ mV	($10^{-1}$ mV)${}^{2}$	mV	$10^{-1}$ mV	($10^{-1}$ mV)${}^{2}$	mV	$10^{-1}$ mV	($10^{-1}$ mV)${}^{2}$
1	182.4	0	0	−103.8	0	0	9.0	−3	9
2	182.6	2	4	−103.9	1	1	9.2	−1	1
3	182.2	−2	4	−104.0	2	4	9.3	0	0
4	182.1	−3	9	−103.7	−1	1	9.6	3	9
5	182.7	3	9	−103.6	−2	4	9.4	1	1
mean	182.4			−103.8			9.3		
SSD			26			10			20

Deviations are calculated as: $\Delta E_{1}\left(i\right)=E_{1}\left(i\right)-\bar{E_{1}}$; $\Delta E_{2}\left(i\right)=E_{2}\left(i\right)-\bar{E_{2}}$; $\Delta E_{X}\left(i\right)=E_{2}\left(i\right)-\bar{E_{X}}$, where $\bar{E_{1}}$, $\bar{E_{2}}$, and $\bar{E_{X}}$ are the mean values of $E_{1}$, $E_{2}$, and $E_{X}$, respectively, and are calculated according to Equation (7). The sums of squared deviations (SSD) are calculated as $\sum_{i}{\left[\Delta E_{n}\left(i\right)\right]}^{2}$ for n=1,2,⋯,X.

**Table 2:** Uncertainty budget for the pH measurement.

Name	Quantity	Estimate	Standard Uncertainty	Sensitivity Coefficient	Uncertainty Contribution	Squares of Uncert. Contr.
Symbol	$X_{i}$	$x_{i}$	$u(x_{i})$	$c_{i}$	$u_{i}\left(y\right)=u\left(x_{i}\right)\cdot c_{i}$	$u_{i}^{2}\left(y\right)$
Column No.	(1)	(2)	(3)	(4)	(5) = (3)·(4)	(6) = ${\left(5\right)}^{2}$
	$E_{1}$	182.4	0.207	0.00690	0.00143	0.0000020
Sources	$E_{2}$	−103.8	0.187	0.0107	0.00198	0.0000039
	$E_{X}$	9.3	0.200	−0.0175	−0.00349	0.0000122
	${\mathrm{pH}}_{1}$	4	0.0289	0.395	0.01142	0.0001304
	${\mathrm{pH}}_{2}$	9	0.0289	0.605	0.01748	0.0003055
Result	${\mathrm{pH}}_{X}$	7.02411			0.02131	0.0004541

**Table 3:** Comparison of simulation times.

Language	Time (s)
C++	6.16
Octave	1.68
R	4.72
Python	3.90

## Abbreviations

The following abbreviations are used in this manuscript:

GUM	Guide to the expression of Uncertainty in Measurement
ISE	Ion-Selective Electrode
MC	Monte Carlo
PDF	Probability Density Function
VIM	International Vocabulary of Metrology
UI	User Interface

## References

[1] Stebel K., Choinski D. (2015). Performance Improvement for Quasi Periodical Disturbances in pH Control. Adv. Electr. Comput. Eng..

[2] JCGM (2008). Evaluation of Measurement Data—Guide to the Expression of Uncertainty in Measurement. https://www.bipm.org/utils/common/documents/jcgm/JCGM_100_2008_E.pdf.

[3] EURACHEM/CITAC (2012). QUAM:2012.P1 Guide CG 4. Quantifying Uncertainty in Analytical Measurement. https://www.eurachem.org/images/stories/Guides/pdf/QUAM2012_P1.pdf.

[4] Amman D. (1986). Ion-Selective Microelectrodes.

[5] Morf W.E. (1981). The Principles of Ion-Selective Electrodes and of Membrane Transport.

[6] Buck R.P., Rondinini S., Covington A.K., Baucke F.G.K., Brett C.M.A., Camoes M.F., Milton M.J.T., Mussini T., Naumann R., Pratt K.W. (2002). Measurement of pH. Definition, standards, and procedures (IUPAC Recommendations 2002). Pure Appl. Chem..

[7] Wiora J. (2016). Problems and risks occurred during uncertainty evaluation of a quantity calculated from correlated parameters: A case study of pH measurement. Accredit. Qual. Assur..

[8] Meinrath G., Spitzer P. (2000). Uncertainties in Determination of pH. Microchim. Acta.

[9] Buchczik D., Ilewicz W. Evaluation of calibration results using the least median of squares method in the case of linear multivariate models. Proceedings of the 2016 21st International Conference on Methods and Models in Automation and Robotics (MMAR).

[10] JCGM (2012). International Vocabulary of Metrology—Basic and General Concepts and Associated Terms (VIM). https://www.bipm.org/utils/common/documents/jcgm/JCGM_200_2012.pdf.

[11] European Accreditation (2013). EA-4/02 Expression of the Uncertainty of Measurement in Calibration.

[12] Rubinson K.A., Rubinson J.F. (2000). Contemporary Instrumental Analysis.

[13] Midgley D., Torrance K. (1991). Potentiometric Water Analysis.

[14] Eckschlager K. (1969). Errors, Measurement and Results in Chemical Analysis.

[15] Wiora J. (2014). Improvement of measurement results based on scattered data in cases where averaging is ineffective. Sens. Actuator B-Chem..

[16] Sharaf M.A., Illan D.L., Kowalski B.R. (1986). Chemometrics; Chemical Analysis.

[17] Fischer H. (2011). A History of the Central Limit Theorem.

[18] Wiora J., Kozyra A., Wiora A. (2016). A weighted method for reducing measurement uncertainty below that which results from maximum permissible error. Meas. Sci. Technol..

[19] Olsen R.J., Sattar S. (2013). Measuring the Gas Constant R: Propagation of Uncertainty and Statistics. J. Chem. Educ..

[20] Kušnerová M., Valíček J., Harničárová M., Hryniewicz T., Rokosz K., Palková Z., Václavík V., Řepka M., Bendová M. (2013). A Proposal for Simplifying the Method of Evaluation of Uncertainties in Measurement Results. Meas. Sci. Rev..

[21] Wiora J., Filev D., Jabłkowski J., Kacprzyk J., Krawczak M., Popchev I., Rutkowski L., Sgurev V., Sotirova E., Szynkarczyk P., Zadrozny S. (2015). Uncertainty Evaluation of pH Measured Using Potentiometric Method. Intelligent Systems’2014.

[22] JCGM (2008). Evaluation of Measurement Data—Supplement 1 to the “Guide to the Expression of Uncertainty in Measurement”—Propagation of Distributions Using a Monte Carlo Method. https://www.bipm.org/utils/common/documents/jcgm/JCGM_101_2008_E.pdf.

[23] American Association for Laboratory Accreditation (2014). G104—Guide for Estimation of Measurement Uncertainty In Testing.

[24] Douglas J.E. (1992). Data analysis in the computer age: A Monte Carlo computer simulation of error sensitivity in the determination of formation constants. J. Chem. Educ..

[25] Theodorou D., Meligotsidou L., Karavoltsos S., Burnetas A., Dassenakis M., Scoullos M. (2011). Comparison of ISO-GUM and Monte Carlo methods for the evaluation of measurement uncertainty: Application to direct cadmium measurement in water by GFAAS. Talanta.

[26] Harris P.M., Cox M.G. (2014). On a Monte Carlo method for measurement uncertainty evaluation and its implementation. Metrologia.

[27] Horne K., Fleming A., Timmins B., Ban H. (2015). Monte Carlo uncertainty analysis for photothermal radiometry measurements using a curve fit process. Metrologia.

[28] Cho C., Lee J.G., Kim J.H., Kim D.C. (2015). Uncertainty Analysis in EVM Measurement Using a Monte Carlo Simulation. IEEE Trans. Instrum. Meas..

[29] Sobol I.M. (1994). A Primer for the Monte Carlo Method.

[30] Gedam S.G., Beaudet S.T. Monte Carlo simulation using Excel(R) spreadsheet for predicting reliability of a complex system. Proceedings of the Reliability and Maintainability Symposium.

[31] Decker J.E., Eves B.J., Pekelsky J.R., Douglas R.J. (2011). Evaluation of uncertainty in grating pitch measurement by optical diffraction using Monte Carlo methods. Meas. Sci. Technol..

[32] Yanai R.D., Battles J.J., Richardson A.D., Blodgett C.A., Wood D.M., Rastetter E.D. (2010). Estimating Uncertainty in Ecosystem Budget Calculations. Ecosystems.

[33] Wiora J. (2010). The Application of a Spreadsheet in the Uncertainty Evaluation Using Monte Carlo Method in the Case of Ion-Selective Electrodes; VIII Polska Konferencja Chemii Analitycznej. https://http://www2.chemia.uj.edu.pl/pkca2010/doc/program.pdf.

[34] Chew G., Walczyk T. (2012). A Monte Carlo approach for estimating measurement uncertainty using standard spreadsheet software. Anal. Bioanal. Chem..

[35] Zangl H., Hoermaier K. (2017). Educational aspects of uncertainty calculation with software tools. Measurement.

[36] Microsoft Excel Function Translations. http://www.computerhope.com/issues/ch000704.htm.

[37] List of Uncertainty Propagation Software. http://en.wikipedia.org/wiki/List_of_uncertainty_propagation_software.

[38] Metrodata GmbH. http://www.metrodata.de.

[39] Qualisyst Ltd.. http://www.qsyst.com.

